# To select or be selected – gendered experiences in clinical training affect medical students’ specialty preferences

**DOI:** 10.1186/s12909-018-1361-5

**Published:** 2018-11-19

**Authors:** Emelie Kristoffersson, Saima Diderichsen, Petra Verdonk, Toine Lagro-Janssen, Katarina Hamberg, Jenny Andersson

**Affiliations:** 1 Department of Public Health and Clinical Medicine, Family Medicine, Umeå University, 901 87 Umeå, Sweden; 2 Umeå Centre for Gender Studies, Umeå University, 901 87 Umeå, Sweden; 3 Department of Medical Humanities, EMGO Institute for Health and Care Research, School of Medical Sciences, VU University Medical Center, Amsterdam, The Netherlands; 4 Department of Primary and Community Care, unit for Gender and Women’s Health, Radboud University Medical Centre, Nijmegen, The Netherlands

**Keywords:** Medical students, Specialty preference, Professional identity formation, Sexism, Mixed methods

## Abstract

**Background:**

The literature investigating female and male medical students’ differing career intentions is extensive. However, medical school experiences and their implications for professional identity formation and specialty choice have attracted less attention. In this study we explore the impact of medical school experiences on students’ specialty preferences, investigate gender similarities and differences, and discuss how both might be related to gender segregation in specialty preference.

**Methods:**

In a questionnaire, 250 Swedish final-year medical students described experiences that made them *interested* and *uninterested* in a specialty. Utilizing a sequential mixed methods design, their responses were analyzed qualitatively to create categories that were compared quantitatively.

**Results:**

Similar proportions of women and men became interested in a specialty based on its knowledge area, patient characteristics, and potential for work-life balance. These aspects, however, often became secondary to whether they felt included or excluded in clinical settings. More women than men had been deterred by specialties with excluding, hostile, or sexist workplace climates (W = 44%, M = 16%). In contrast, more men had been discouraged by specialties’ knowledge areas (W = 27%, M = 47%).

**Conclusions:**

Male and female undergraduates have similar incentives and concerns regarding their career. However, the prevalence of hostility and sexism in the learning environment discourages especially women from some specialties. To reduce gender segregation in specialty choice, energy should be directed towards counteracting hostile workplace climates that explain apparent stereotypical assumptions about career preferences of men and women.

## Background

Despite a preponderance of women in medical schools there remains an unequal distribution of men and women over specialties and in medical leadership [1, 2]. A stable pattern of gender segregation in specialty preferences has been observed already among undergraduate medical students in many countries [3]. The driving forces behind this segregation is often suggested to be that women strive for a balance between work and family life, while men desire technical challenges, salary, career prospects, and prestige [4–8]. However, most previous studies have relied on questionnaires offering respondents fixed alternatives with pre-formulated motivational factors, while omitting students’ own descriptions of what they consider crucial in their specialty considerations. Furthermore, researchers have suggested that insights about the relative importance of [9], and interrelations between different predictors/motivational factors [10], are lacking.

In parallel, there is research showing how experiences during clinical training, such as role modeling or exposure to harassment, affect students’ sense of belonging and (dis)identification on different wards, thus driving them to avoid particular specialties [11–16]. Most of these studies have been conducted solely among female students or have explored interest in specific specialties. Still, they indicate a process whereby students seek coherence between their professional self-perceptions and how supervisors treat and recognize them, and the importance of feeling welcome and accepted in a specialty.

Inclusion is also fundamental to professional identity formation [17]. In order to develop into a capable and confident professional, it is necessary to experience oneself as an accepted and competent individual within one’s medical community [18–20]. Through observation and interaction with supervisors and other role models students learn professional values and norms, and adopt them in order to establish legitimacy as physicians-to-be [21, 22]. Thus, students not only incorporate or resist values and principles advocated in formal curricula. They also incorporate norms, attitudes and behaviors conveyed by supervisors and staff in everyday clinical work – often referred to as the informal or hidden curriculum [23]. It might also be called the gendered hidden curriculum as professional standards in medicine have been shown to still adhere to traditional masculine norms of objectivity, detachment, authority, and competition rather than involvement, cooperation, and empathy [21, 24, 25].

Attitudes towards gender in the educational environment have important implications for medical students’ clinical experiences. Female students are more likely to encounter sexism such as gender related prejudice, gender discrimination, and sexual harassment, than are their male peers [16, 26, 27]. Learners’ ways of being and acting are contested or confirmed by gender-related expectations and evaluations from staff and supervisors, affecting how they interpret their role as future physicians [11, 13, 26]. Even subtle interactional processes of inclusion and exclusion on the basis of gender, e.g. sexist jokes, diminishing, and ridicule, convey messages to people about the rightful place of women and men in medicine [12]. Yet, we do not fully comprehend how these unequal experiences shape the subsequent specialty preferences of students.

Researchers have suggested that the cumulative effect of overt and covert forms of sexism in the learning environment create an adverse climate for female students that dampens their confidence, participation, and aspirations [12, 28, 29]. Subtle inequities, through which women are treated differently, have also been termed ‘everyday sexism’ [12] or ‘gender microaggressions’ [29]; verbal and nonverbal slights that intentionally or unintentionally convey disregard or contempt. Although such discrimination can be both unintentional and inconspicuous, they are manifestations of worldviews of gender superiority/inferiority [29]. Despite societies or organizations, e.g. medical schools, formal commitments to gender equality, inequities become part of everyday life and are often perceived as “normal” and therefore are not recognized [12, 28, 29]. Thus, to further explore the identification process which links clinical experiences to professional identity and career intentions, we need to consider the possibility of overt and subtle forms of sexism in clinical training settings.

In a questionnaire to Swedish final year medical students we included open-ended questions about concrete experiences that made them interested versus uninterested in a specialty. Our aim was to explore the impact of medical school experiences on students’ specialty preferences, to investigate similarities and differences between men and women regarding the character and consequences of experiences described, and to discuss how this might be related to gender segregation in specialty preference.

## Methods

### Study design

This study is part of the project ‘Gender Challenges in Medical Education’, aimed at investigating medical students’ attitudes and thoughts about gender-related questions [30]. A study design deploying open-ended questions in a survey, analyzed with a mixed methods approach [31], was chosen in order to enable exploration of individual experiences and comparisons between groups. We decided to confine our study to final year students since they were most likely to be reflecting on their study period and their forthcoming specialty choices.

### Setting

The study was conducted at the medical school at Umeå University in Northern Sweden. In Sweden, paid parental leave and subsidized childcare encourages women and men to share paid and unpaid work [32]. At present, women constitute more than half of Swedish medical students and physicians [2, 33]. However, as in other countries, gender differences in specialty choices are common with male dominated specialties holding higher status and/or salaries [2, 34].

Undergraduate medical education in Sweden comprises 5.5 years, including 3 years of clinical training. The undergraduate period is followed by an 18–24 month internship that is required before applying for a residency position.

During the clinical phase of their program, medical students in Umeå rotate between different wards at the university hospital, local hospitals, and health care centers in the region. The proportion of female medical students in Umeå has been 50–60% in the past 10 years. According to questionnaires administered to all students in 2006–2009 a large majority came from a middle class background and less than 10% had parents who were born outside of Scandinavia [35].

### Data collection and participants

Between 2011 and 2013, final-year medical students at Umeå University were invited to respond to the anonymous questionnaire “Gender in medical education”. After demographic queries, the survey continued with two open-ended questions*:* “Can you describe an event that made you *interested* in working in a certain specialty?” and: “Can you describe an event that made you *uninterested* in working in a certain specialty?”. Subsequent questions and scales measuring respondents’ attitudes to gender issues in medicine are not included in this analysis.

Students were informed about the study when attending compulsory lectures, unrelated to the study, and participation was voluntary. No attempt was made to reach students who did not attend the lecture where the questionnaire was administered. The lecture, however, was mandatory and we have no reason to believe that the absent students differed in any specific way from those present.

Out of 404 students (227 women, 56%) registered during the study period, 344 students were present at the lectures and therefore invited, and 305 of them stayed on to fill out the questionnaire. After exclusion of 55 students who did not answer either of the two open questions 250 remained (146 women, (58%), 104 men), giving a response rate of 73%.

The Regional Ethics Committee in Umeå approved the study: (dnr 2011–262-31 M.). The return of students’ completed questionnaires was considered as consent for participation.

### Quantitative analysis of socio-demographics

Pearson’s chi-square test was used for comparing socio-demographics for responders and non-responders. A *p*-value < 0.05 was considered significant.

### Mixed methods analysis of the open-ended answers

We adopted a simple sequential mixed-method design to explore students’ experiences and observe similarities and differences between men and women [31]. It was sequential in the sense that in a first phase the students’ handwritten answers were transcribed and analyzed by way of qualitative content analysis to elaborate categories [36]. In a second phase, the sizes of the categories were analyzed quantitatively and the proportion of answers from men and women in each category was compared.

#### Phase 1: Elaborating categories

Prior to the analysis, students’ responses were ‘blinded’ from socio-demographic information, e.g. gender. However, a few participants referred to themselves as either a man or a woman in a way that was not possible to blind. The last author (JA) read one third of the answers to get an overview of the content, conducted an open coding and created a preliminary coding schedule. Answers from 100 students were then read, coded and categorized independently by the four Swedish-speaking authors (EK, SD, KH, JA). In joint sessions, the coding and labeling of categories was then compared, discussed, and reformulated. Based on this, answers from another 50 students were read, categorized, and discussed. This procedure was repeated one more time, at which point the elaborated categories seemed consistent and varied enough to capture the variety of answers. In this way a final coding schedule of eight categories was established. Thereafter, the first author (EK) reread and encoded all answers, i.e., conducted the main coding. To facilitate understanding and presentation, related categories were grouped into three themes.

To check the reliability of the main coding, two of the authors (KH, JA) individually encoded 160 randomly chosen answers (80 respectively about experiences inducing interest and disinterest). Each answer was encoded according to the categories in the final coding schedule and the codings were then compared to the first authors coding. A total of 68 discrepancies were identified and these were spread among authors as well as answers about interest and disinterest. With three authors (main coder EK, KH, JA), 160 answers, and eight categories the percentage of discrepancies was less that 2% (68/(160 × 8 × 3) = 0,018). Thus, the inter-rater reliability test showed high consensus, suggesting that the categories were clear and consistent.

#### Phase 2: Quantitative analysis

When the main coding was completed and all answers sorted into categories, we created a chart in SPSS (version 24.0 for Mac OSX). If a category was coded in an answer it was marked with ‘1’, otherwise with ‘0’. The proportions of answers coded with ‘1’ within each category were compared between inspiring and deterring experiences, and between men and women, and tested using the Pearson chi-square test. A *p*-value < 0.01 was considered significant. The number of words in the answers was also counted and compared between men and women (range and mean).

## Results

Below we present students’ socio-demographics, followed by the themes and categories developed in the qualitative analysis of free text answers. Finally, we present the statistical analysis of gender patterns in the categories.

### Socio-demographics

Men and women had similar socio-demographics, as shown in Table 1. A vast majority of the students were born in Sweden and had highly educated parents. Most self-identified as heterosexual and few had children. The 55 excluded students did not differ significantly from the participants (not presented in Table 1).

**Table 1:** Socio-demographics of the 250 included female and male students

Category	Variable	All (*N* = 250)	Women *N* = 146 (58%) Mean (SD)	Men *N* = 104 (42%) Mean (SD)
Age		Range: 23–55 Mean: 28.33 SD: 4.32 Median: 27	Range: 23–55 Mean: 28.03 SD: 4.45 Median: 27	Range: 23–49 Mean: 28.74 SD: 4.13 Median: 28
Civil status	Not cohabiting	102 (40.8)	58 (39.7)	44 (42.3)
	Cohabiting/married	148 (59.2)	88 (60.3)	60 (57.7)
Children	No	214 (85.6)	127 (87.0)	87 (83.7)
	Yes	36 (14.4)	19 (13.0)	17 (16.3)
Sexual orientation	Heterosexual	235 (94.0)	135 (92.5)	100 (96.2)
	Homo, bi, or queer	15 (6.0)	11 (7.5)	4 (3.8)
Country of birth	Sweden	233 (93.2)	138 (94.5)	95 (91.3)
	Other than Sweden	17 (6.8)	8 (5.5)	9 (8.7)
Parents’ country of birth other than Sweden	Both	18 (7.2)	9 (6.2)	9 (8.7)
	One	16 (6.4)	12 (8.2)	4 (3.8)
	None	216 (86.4)	125 (85.6)	91 (87.5)
Parents with higher education^a^	Both	167 (66.8)	97 (66.4)	70 (67.3)
	One	52 (20.8)	34 (23.3)	18 (17.3)
	None	30 (12.0)	14 (9.6)	16 (15.4)
Parents’ occupation physician	Both	6 (2.4)	3 (2.1)	3 (2.9)
	One	50 (20.0)	32 (21.9)	18 (17.3)
	None	194 (77.6)	111 (76.0)	83 (79.8)

^a^Answer from one female student missing

### Themes and categories in the free text answers

The answers varied in level of detail and depth. Most responses coded for more than one category. The results comprised three themes; ‘The character of work suits me’, ‘Inspiring and inclusive workplace’, and ‘Matches my work-life priorities’. Each theme consists of two to three categories. The content and examples of all categories are outlined in Table 2.

**Table 2:** Themes, categories, category descriptions and examples of quotes included in each category

THEME/category	Description	Quotes interested	Quotes uninterested
The character of work suits me			
Knowledge area and practice	Area of knowledge, routine work or specific work tasks. Own talent, previous work, studies, research or personal experiences.	“I felt like I had a talent for image interpretation”, “my interest in geriatrics began when I started my first job as a nursing assistant”	“Endless rounds where the question is half a pill here or there”, “seems boring to attach bowels for hours”, “it was smelly and slimy”
Patient characteristics and patient contact	Patient groups perceived as ungrateful or rewarding to work with. Amount of patient contact, variation and continuity. Memorable patient encounters.	“I got a lot back from the patients, even though it was often burdensome”, “restoring a fractured wrist and immediately seeing results”.	“large proportion of unmotivated patients”, “old patients with multi morbidity”
Values and care ideology	Perceptions of whether care ideology in a specialty is in alignment with personal values, e.g. how patients are treated. Also whether the job would enable students to fulfill own work priorities, e.g. work for the underprivileged.	“holistic approach to patients”, “I have travelled to underserved areas and seen the needs”.	“too much time spent on craftsmanship and too little time talking to patients”
Inspiring and inclusive workplace			
Workplace climate	General impressions of workplace climate and attitudes; how physicians treat each other and other staff, communicate or work together.	“friendly workgroup”, “staff who seemed to work as a team”, “supportive colleagues”	“endless days in the operating room listening to macho jargon”, “very bad atmosphere among the physicians on the ward”
Supervision and participation	Treatment of students at the clinic; reception, introduction, supervision, feedback practices, and amount of participation.	“including reception”, “student out-patient clinic with engaging supervisor”, “got a lot of praise and felt good”	“unpleasant treatment from male physicians”, “I was placed at the patient’s feet and saw nothing. No one bothered to explain either”
Role models	Physician role models to strive after, identify with, or distance from: empathic and devoted, or cold hearted and cynical.	“a talented physician that had an amazing relationship with the patients”, “the physician stayed calm in a chaotic situation”	“a consultant who humiliated the patient”, “a surgery that went wrong, and the way the three surgeons involved tried to resign from responsibility”
Matches my work-life priorities			
Workload	Workload in relation to personal circumstances and life style. Working hours, on-call, workload and stress. How the clinic function in terms of leadership, staffing situation and overtime.	“I finished at 16:00 – there were time for other things in life as well!”, “being able to control your own time”	“you have to sacrifice everything else in life”. “being expected to work extra in the evening for free”, “heavy workload and unreasonable demands”
Development possibilities	Opportunities for professional development or research.	“A challenge to work with your own preconceptions and communication skills”	“It takes years before you are somewhat autonomous”

In our presentation of themes, more space is given to the second theme ‘Inspiring and inclusive workplace’. The reason is that this theme in particular conveyed examples of students’ educational experiences and the relational settings in which professional identity formation is situated. The answers within the other two themes were often shorter, included less variation, and conveyed simple arguments and views rather than accounts of concrete experiences. Still all three themes are important and were often interconnected.

In the excerpts, details about wards and staff have been modified to ensure confidentiality. For example, quotes pertaining to specific surgical specialties e.g.: ‘general surgery’, ‘orthopedics’, etc., have been changed to ‘surgery’ or ‘surgical specialty’.

#### ‘The character of work suits me’

*‘Knowledge area and practice’:* Participants recounted specific work tasks that intrigued them or matched their perceived talent, like ‘the discussions’ and the ‘detective work’ of internal medicine, or the surgical ‘craftsmanship’. Interest in specific work tasks, however, was often initiated, or maintained by invitations to hands-on participation:

I was interested in surgery before I started medical school, but what kept me interested was that I got the opportunity to try a lot on my own. (woman)

Correspondingly, being a passive bystander rendered the same knowledge area or work assignments being labeled as ‘boring’, ‘monotonous’, and ‘deterring’.

*‘Patient characteristics and patient contact’:* Rewarding patients were portrayed as grateful, or ‘giving a lot back’ – spanning from particular age groups, like children and elders to people with concrete problems that could be cured or ‘fixed’. Meeting patients with severe or incurable diseases, or trauma victims, could also make a lasting impression.

Conversely, patients suffering from ‘multiple morbidities’, diffuse symptoms, or those who were ‘unmotivated’ or ‘ungrateful’ were discouraging for some students:

When I met a patient with chronic pain, who had very little drive and who was totally uninterested in working to reach forward. For some reason, this provoked me. (man)

*‘Values and care ideology.’* Some participants considered whether they shared values and care ideology (e.g. patient-centered care) with physicians in a particular specialty. Others wanted to make a difference by working in medically underserved areas of the world. Workplaces where values were at odds with those of the student were described as dissuading:

I lose interest when all that matters is how fast you finish off the operation list - not how well you take care of the patients. (w)

#### ‘Inspiring and inclusive workplace’

*‘Workplace climate’:* Often participants explained that even though patients and work tasks were important, the workplace climate was paramount when opting for a specialty:

The clinic and my co-workers will be of greater importance than the work itself. (w)

Students were attracted by nonhierarchical wards, well functioning cooperation, and staff who seemed to enjoy their job and supported each other – factors that often were interconnected. However, workplace climates, characterized as ‘hierarchical’ and ‘tough’, and with senior physicians who treated colleagues of lower rank poorly were described as reasons for not choosing a certain specialty. Most examples concerned male-dominated and surgical wards where students also noted prevalence of ‘abuse of power’, ‘sexist jokes’, ‘macho jargon’, and ‘macho attitudes’; behaviors with which they disagreed:

Chauvinistic and macho atmosphere reoccurred on morning rounds. Sexist jokes were told and I lost interest completely for that clinic. I just couldn’t stand it there. (w)

There were also negative accounts of competitive rather than supportive attitudes, as well as accusations and conflict rather than solidarity where ‘not showing weakness’ and ‘not asking for help’ were encouraged. Likewise, settings where residents were expected to be wholly devoted to work and have no other interests were deterring.

Some students disclosed that experiencing a bad environment compelled them to revise previous career plans:

Since I have seen what the jargon is like at several surgical clinics and how unpleasant many surgeons are, I am no longer interested in working there although I still think it is an exciting subject. (m)

Overall, accounts of workplace climate did not explicitly concern gender, although ‘macho jargon’ and ‘sexist jokes’ was mentioned. Still, some women overtly discussed gender related issues. Seeing specific challenges facing women in male-dominated clinics, for example difficulty gaining respect, worried them:

The only female physician expressed her disappointment over that the other physicians did not prioritize the ward. The atmosphere became very tense, and she got very little support. I do not want to work as the sole female physician in my team. (w)

*‘Supervision and participation’:* Participants’ interest was spurred by experiences of being seen, included, and taken seriously by supervisors:

I had good contact with a supervisor who gave me the “right” space to work independently, combined with good support. As a result I had fun during my placement and thus aroused interest in the specialty. (w)

Positive attention, like constructive feedback and praise, or being offered a job, made long lasting impressions. Active participation, and getting to do practical tasks were also important. This student described his overwhelming first experience in the operating room:

I went from being totally uninterested to being quite interested in surgery the first day of the clinical placement. I got to assist on a surgery and meanwhile I got fantastic tutoring from the surgeon. (m)

Lack of supervision or participation on the other hand made a negative impression, as did being ‘used as free labor’, or feeling ‘excluded’ or ‘ignored’:

I lost interest already at the first morning meeting when all the surgeons treated us students like we were invisible, and also ignored pretty much everything the interns and everyone else said. (m)

Some respondents disclosed how they had been subjected to domination techniques, or felt humiliated by supervisors. Such treatment had the power to literally ‘destroy’ clinical placements. Experiences of neglect were also detrimental and some students, particularly women, described such incidents as gender related:

I lose interest when I feel spoken to as if I'm not a potential future colleague at work - usually from a male physician. (w)

*‘Role models’:* Role models depicted as caring and empathic towards patients and students, who seemed enthusiastic and dedicated even after many years within the profession, were highlighted as important for specialty preferences:

When I meet an experienced physician that is still excited about patients and being a supervisor. (m)

Negative role models in contrast were those who demonstrated cynicism and detachment towards their work. They belittled students and staff, and tried to escape their responsibilities as physicians and supervisors. Other accounts pertained to physicians who treated patients insensitively and harshly:

When a surgeon during the morning rounds in an abstruse and cold-hearted way notified a patient that he had cancer, a patient who had lost his wife in cancer a couple of years earlier. (w)

#### ‘Matches my work-life priorities’

*‘Workload’:* Working office hours and being in control of one’s work schedule, often related to the potential of combining paid work with family and leisure time, was generally considered preferable. Poor organization, heavy workloads, and seeing residents and consultants staying on past regular hours, and working nightshifts scared the students:

It doesn’t matter how interesting a specialty is, if I have to be awake at night I will not be able to appreciate it. I don’t want to have to ‘live through’ a shift. I want each day to be enjoyable and rewarding. (w)

*‘Development possibilities’:* Some accounts pertained to the potential for professional development in a specialty, for example, ‘platforms for improving communication skills’, ‘promising technical innovations’, and research possibilities. Some also gave negative reports of ‘scarce development possibilities’ or ‘expectations to work without pay’.

### Gender similarities and differences

**Fig. 1:**
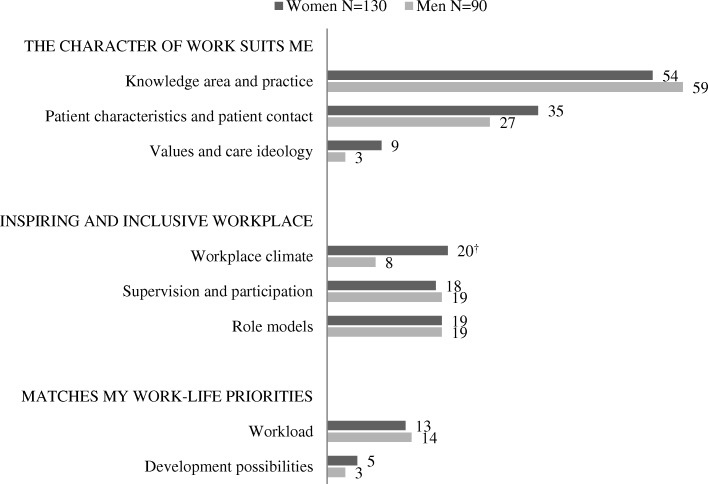
Experiences inducing *interest* in a specialty. Proportions (%) of answers from male and female students represented in each category #. One answer could include information belonging to more than one category. # Among the 250 students (146 women, 104 men), 220 described events inducing interest (130 women, 90 men) for a specialty. *p* values calculated using Chi-Square test, the level of significance was set to 0.01. All differences between women and men were non-significant. ^†^ Close to significant (*p* = 0.013)

**Fig. 2:**
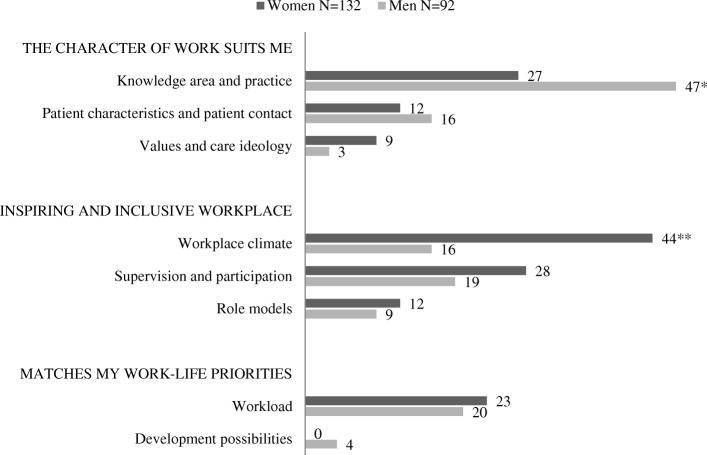
Experiences inducing *uninterest* in a specialty. Proportions (%) of answers from male and female students represented in each category #. One answer could include information belonging to more than one category. Among the 250 students (146 women, 104 men), 224 described events inducing non-interest (132 women, 92 men) for a specialty. p values calculated using Chi-Square test, the level of significance was set to 0.01. * *p* < 0.01; ** *p* < 0.001

Most participants (about 90%, men and women alike) responded to both of the open-ended questions, resulting in 220 answers regarding experiences inducing interest and 224 answers about experiences inducing non-interest (Figures 1&2, comment at the bottom). Overall, women’s answers were 30 words long (Range; 3–116), and covered 2.9 categories. Men’s answers were on average 20 words long (Range; 2–74), and covered 2.5 categories. The distributions of the categories in male and female students’ answers are presented separately for experiences inducing interest (Fig 1) and non-interest (Fig 2).

Men and women showed a similar pattern with no significant differences found when comparing the relative proportions of men and women describing experiences that fostered interest in a specialty (Fig 1). Examples regarding ‘Knowledge area and practice’ were the most common (W = 54%, M = 59%), and ‘Patient characteristics and patient contact’ the second most common (W = 35%, M = 27%). However, a greater proportion of women than men described that they had become interested in a specialty after experiencing a good ‘Workplace climate’ (W = 20%, M = 8%), and this difference was close to significant (*p* = 0.013). Workplace climate was the third most common incentive for women. Among men, the third most prevalent incentives were ‘Supervision and participation’ and ‘Role models’ (both M = 19%). Compared with men, women gave longer answers including more categories. As a result, even though ‘Supervision and participation’ and ‘Role models’ did not rank among the top three incentives among women, they still occurred just as frequently as for men (at 18 and 19% respectively).

Gender patterns were more disparate for deterrent experiences (Fig 2). The largest difference was seen in the category ‘Workplace climate’. This category was the most common deterrent among women, but did not even rank among the top three deterrents for men (W = 44%, M = 16%, *p* < 0.001). There was also a significant gender difference in the category ‘Knowledge area and practice’ which was the most common deterrent among men but the third most common deterrent for women (W = 27%, M = 47%, *p* < 0.01). The category ‘Supervision and participation’ ranked second (at 28%) among women’s deterring experiences, followed by ‘Workload’ (W = 23%). Among men, the second and third most common deterrents were ‘Workload’ (M = 20%), closely followed by ‘Supervision and participation’ (M = 19%).

## Discussion

We explored how medical students’ specialty preferences are shaped by educational experiences, and investigated similarities and differences between men and women regarding the character and consequences of experiences described. Male and female participants shared common inspiring experiences pertaining to knowledge area, their own talent, and patient groups. However, participants’ impressions of workplace climate, supervision, and participation were often described to be of greater importance in their specialty considerations. These experiences rendered feelings of inclusion or exclusion and sometimes outweighed other experiences during clinical practice. Among women, the most common reason for avoiding a specialty was related to experiences of hierarchical, hostile, and sexist workplace climates. In contrast, perceived lack of interest in the knowledge area of a given medical field was the most common reason for men to avoid a specialty.

### To select or be selected

Earlier studies have showed that it is mainly women who consider patient orientation, while men focus on technical challenges [4–8]. By exploring both encouraging and discouraging experiences our results add nuance to this picture, showing gender similarities in inspiring experiences of patient groups and knowledge areas. However, despite interest in the knowledge area of particular specialties, participants were willing to revise their career plans if they encountered negative/discouraging workplace climates. Thus, answering the call from Querido et al. [10] to explore interrelations among predictors of specialty choice; our results suggest that the workplace climate, which tended to be more adverse for the women, had high relative impact and outweighed other motivational factors. Consequently, this study contributes to the discussion on the process of gender segregated career decisions.

Even if specialty preference at first seemed to be about students selecting it was just as much a matter of feeling selected and welcomed at a workplace. Knowledge area was the most common factor that made students, women and men alike, interested in a specialty. However, the fact that more women than men described experiences of a hostile and unwelcoming workplace climate made it more of a male privilege to choose a specialty according to interest in its knowledge base. In keeping with previous research, there were more examples of unwelcoming workplace climates from male-dominated and surgical specialties [16, 27]. Thus, our results make a valuable contribution to understanding how subtle inequities perpetuate the pattern of gender segregation in specialty preference.

A sexist jargon and difficulties for women gaining respect described by our participants, convey the message that women are less worthy and less capable than men [28, 29]. It is reasonable to suggest that the cumulative effect of those minor slights hampered female students’ confidence and career aspirations. In concurrence with other studies [13, 16], our findings showed that even without personal experiences of abusive treatment specialty considerations could be affected. Observing others being mistreated, or overhearing subtle manifestations of sexism, can be damaging enough. Descriptions of an inclusive workplace climate were also more prevalent in women’s’ encouraging experiences, although this difference was not significant, suggesting a positive reception was not taken for granted and therefore noted and valued by more women than men. It is well known that female students are exposed to more discrimination, sexual harassment, and covert sexism [12, 16, 26, 27], and it is likely that this makes them more observant of factors related to workplace climate compared to their male peers.

Publicly espoused professional values in Swedish health care include respect, empathy, and gender equality [37]. Our results, however, conveyed an image of supervisors, at times, acting in direct opposition to official norms. If students could not accept or identify with these behaviors this could be a reason for dismissing a specialty. In accordance with other studies [11, 22], our results indicate that participants accepted and/or adopted the values and behaviors of the group to which they wanted to belong. In clinics where they could not reconcile with the prevailing values, or if they themselves were the victim of professional breaches and/or sexism, they gave up and intended to seek a career elsewhere.

Lave and Wenger [19] propose that whether people incorporate or resist cultural norms depends on which roles they strive for, but also on the positions they are given in a specific context. As shown in our study, even if female and male students might strive for careers in the same specialties, their positions at the clinics differ. Women are more exposed to microaggressions and gendered prejudices at the clinics, which probably makes acculturation difficult for them. In contrast, due to their relatively privileged position, male students are more likely to remain oblivious to gendered power dynamics and sexist workplace climates, making acculturation less complicated. Alternatively, male students may be equally aware of hostile climates but not as troubled by them. Still, an important finding in our study was that also a substantial proportion of the male students had noted and become deterred by working climates described as hostile and hierarchical.

Another example of resignation concerned work-life priorities. Our students were dissuaded from specialties where they saw residents struggling with high workloads and expectations to prioritize work above all else. Work-life balance is often suggested to be an issue for women [4, 5, 8], but in line with some previous reports, proportions of men and women being discouraged by specialties with high workload were similar in this study [38–40].

### Implications for medical education

Even if students can discard specialties where supervisors display unprofessional behaviors and sexism they still cannot avoid interaction with these physicians during compulsory clinical training. Although it was primarily women who raised concerns about an adverse workplace climate, quite a few men had similar complaints and changes in the climate would probably benefit the professional development of all students, even those who are not apparently troubled by the problem. By creating welcoming workplace climates, medical schools and other stakeholders in the future medical workforce can therefore provide *all* students with good opportunities to pursue their career goals, and improve their professional development.

However, this might constitute a challenge, as research shows that elusive gendered inequities are often communicated by well-intended people who are unaware of their harmful conduct [28, 29]. The negative impact of these behaviors is usually not recognized by perpetrators or bystanders because they are unaware of the underlying and normalized gender hierarchies [29]. Consequently, there is a need for cooperation between education officials and healthcare professionals to create awareness among students, supervisors and other health care staff about how women and men, overtly or inadvertently, are treated differently in a way that often favors men.

### Methodological considerations, strengths, and limitations

We used free-text answers from a questionnaire, analyzed by means of mixed methods, to explore and compare how male and female medical students experiences from clinical practice might affect their specialty preferences. The use of qualitatively evolved categories grounded in the data when performing statistical analyses helped us highlight what the students themselves chose to focus on instead of using pre-defined categories.

The questionnaire enabled us to represent a large number of students and their multiple realities and to search for gender patterns. The response rate was high and it was similar for women and men, reflecting the distribution of women and men at the Umeå medical school. Although in-depth answers from students were lacking, the answers were given spontaneously by all without having to probe. However, a limitation inherent in using a questionnaire is that it precludes researchers from asking respondents to clarify comments.

In research focusing on differences between women and men there is always a risk of exaggerating gender differences. Thus, the open-ended questions were placed at the beginning of the questionnaire, in order to lower the risk of biasing responses by subsequent questions concerning gender. Furthermore, we did not search for gendered patterns until after categories were outlined, a procedure we believe limited the risk of exaggerating gender differences caused by researchers’ expectations. Finally, since women’s answers were, on average, longer and included more categories than did men’s we considered which categories were the most common in women and men’s answers respectively, rather than focusing only on the gender differences in each category separately.

Our findings are confined to the perceptions of students from one Swedish medical school. Even though Sweden ranks high on international measures of gender equality [41], participants described sexist workplace climates, an image resembling that from studies performed in other countries [27]. Thus, the character of participants’ experiences, their impact on specialty preference and the gender patterns found in our study are probably transferable to other countries.

## Conclusions

(Un)professional beliefs, values, norms, and behaviors among supervisors and staff shape the clinical experiences of medical students, affecting their professional identity formation and how they imagine their future careers. Although male and female undergraduates have similar incentives and concerns regarding career, they enter clinical environments that tend to be more hostile for women, resulting in them feeling less welcomed or even excluded. Irrespective of students’ interests and aptitudes, feelings of inclusion or exclusion in specific workplaces, likely affect subsequent specialty choices. Thus, the understanding of gender as merely a socio-demographic factor, which primes women for ‘family friendly’ specialties and part-time work, and men for technical specialties and prestigious careers, needs to be revised. Future research and debates about gender segregated specialties should be directed towards countering problematic workplace climates that still make it more of a male privilege to choose specialties according to interests.

## References

[1] Kilminster S, Downes J, Gough B, Murdoch-Eaton D, Roberts T (2007). Women in medicine − is there a problem? A literature review of the changing gender composition, structures and occupational cultures in medicine. Med Educ.

[2] Swedish Medical Association. Läkarfakta 2016. Statistik över medlemmar i Sveriges läkarförbund. (Report in Swedish) [Facts about physicians 2016. Statistics on members of the Swedish Medical Association]. https://slf.se/app/uploads/2018/04/laxxkarfakta-2016.pdf. Accessed 14 July 2018.

[3] Alers M, van Leerdam L, Dielissen P, Lagro-Janssen A (2014). Gendered specialities during medical education: a literature review. Perspect Med Educ.

[4] Alers M, Verdonk P, Bor H, Hamberg K, Lagro-Janssen A (2014). Gendered career considerations consolidate from the start of medical education. Int J Med Educ.

[5] Buddeberg-Fischer B, Klaghofer R, Abel T, Buddeberg C (2003). The influence of gender and personality traits on the career planning of Swiss medical students. Swiss Med Wkly.

[6] Cleland JA, Johnston PW, Anthony M, Khan N, Scott NW (2014). A survey of factors influencing career preference in new-entrant and exiting medical students from four UK medical schools. BMC Med Educ..

[7] Lefevre JH, Roupret M, Kerneis S, Karila L (2010). Career choices of medical students: a national survey of 1780 students. Med Educ.

[8] van Tongeren-Alers M, van Esch M, Verdonk P, Johansson E, Hamberg K, Lagro-Janssen T (2011). Are new medical students’ specialty preferences gendered? Related motivational factors at a Dutch medical school. Teach Learn Med.

[9] Cleland JA, Johnston P, Watson V, Krucien N, Skåtun D (2017). What do UK medical students value most in their careers? A discrete choice experiment. Med Educ.

[10] Querido SJ, Vergouw D, Wigersma L, Batenburg RS, De Rond ME, Ten Cate OT (2016). Dynamics of career choice among students in undergraduate medical courses. A BEME systematic review: BEME guide no. 33. Med Teach.

[11] Babaria P, Abedin S, Berg D, Nunez-Smith M (2012). “I’m too used to it”: a longitudinal qualitative study of third year female medical students’ experiences of gendered encounters in medical education. Soc Sci Med.

[12] Beagan B (2001). Micro inequities and everyday inequalities: ‘race’, gender, sexuality and class in medical school. Can J Sociol.

[13] Hill E, Vaughan S (2013). The only girl in the room: how paradigmatic trajectories deter female students from surgical careers. Med Educ.

[14] Nicholson S, Hastings AM, McKinley RK (2016). Influences on students’ career decisions concerning general practice: a focus group study. Br J Gen Pract.

[15] Peel JK, Schlachta CM, Alkhamesi NA (2018). A systematic review of the factors affecting choice of surgery as a career. Can J Surg.

[16] Stratton TD, McLaughlin MA, Witte FM, Fosson SE, Nora LM (2005). Does students’ exposure to gender discrimination and sexual harassment in medical school affect specialty choice and residency program selection?. Acad Med.

[17] Pratt MG, Rockmann KW, Kaufmann JB (2006). Constructing professional identity: the role of work and identity learning cycles in the customization of identity among medical residents. Acad Manag J.

[18] Holden MD, Buck E, Luk J, Ambriz FM, Boisaubin EV, Clark MA (2015). Professional identity formation: creating a longitudinal framework through TIME (transformation in medical education). Acad Med.

[19] Lave J, Wenger E (1991). Situated learning: legitimate peripheral participation. Learning in doing. Cambridge England.

[20] Monrouxe LV (2010). Identity, identification and medical education: why should we care?. Med Educ.

[21] Bleakley A (2013). Gender matters in medical education. Med Educ.

[22] Phillips SP, Clarke M (2012). More than an education: the hidden curriculum, professional attitudes and career choice. Med Educ.

[23] Hafferty FW (1998). Beyond curriculum reform. confronting medicine’s hidden curriculum Acad Med.

[24] Bickel J (2001). Gender equity in undergraduate medical education: a status report. J Womens Health Gend Based Med.

[25] Davies K (2003). The body and doing gender: the relations between doctors and nurses in hospital work. Sociol Health Illn.

[26] Kristoffersson E, Andersson J, Bengs C, Hamberg K (2016). Experiences of the gender climate in clinical training–a focus group study among Swedish medical students. BMC Med Educ..

[27] Fnais N, Soobiah C, Chen MH, Lillie E, Perrier L, Tashkhandi M (2014). Harassment and discrimination in medical training: a systematic review and meta-analysis. Acad Med.

[28] Hall RM, Sandler BR (1982). The classroom climate: a chilly one for women? Project on the status and education of women.

[29] Sue DW (2010). Microaggressions in everyday life: race, gender, and sexual orientation.

[30] Lagro-Janssen A, Verdonk P, Hamberg K, Johansson E (2007). Gender challenges in medical education project. Internal report.

[31] Onwuegbuzie AJ, Tashakkori A, Teddlie C (2003). Teddlie C. a framework for analyzing data in mixed methods research. Handbook of mixed methods in social & behavioral research.

[32] Evertsson M, England P, Mooi-Reci I, Hermsen J, de BJ, Cotter D (2009). Is gender inequality greater at lower or higher educational levels? Common patterns in the Netherlands, Sweden, and the United States. Soc Polit Int Stud Gender, State Soc.

[33] Statistics Sweden. Trender och prognoser 2014 befolkningen, utbildningen, arbetsmarknaden - med sikte på år 2035 (Report in Swedish with English summary) [Trends and forecasts 2014, population, education and labour market in Sweden – outlook to year 2035]. https://www.scb.se/Statistik/_Publikationer/UF0515_2014I35_BR_AM85BR1401.pdf. Accessed 14 July 2018.

[34] Dahl AS. Fortfarande skillnader i lön mellan könen [In Swedish. English title: Still gender differences in physicians salary]. Lakartidningen 2016;113:D4HH. http://www.lakartidningen.se/Aktuellt/Nyheter/2016/05/Fortfarande-loneskillnader-mellan-konen/. Accessed 1 Feb 2018.

[35] Andersson J, Verdonk P, Johansson EE, Lagro-Janssen T, Hamberg K (2012). Comparing gender awareness in Dutch and Swedish first-year medical students - results from a questionaire. BMC Med Educ.

[36] Graneheim UH, Lundman B (2004). Qualitative content analysis in nursing research: concepts, procedures and measures to achieve trustworthiness. Nurse Educ Today.

[37] SFS 2014:821. Patientlag. [In Swedish. English title: Patient Act]. Stockholm: Socialdepartementet. https://www.riksdagen.se/sv/dokument-lagar/dokument/svensk-forfattningssamling/patientlag-2014821_sfs-2014-821. Accessed 31 Jan 2018.

[38] Diderichsen S, Andersson J, Johansson EE, Verdonk P, Lagro-Janssen A, Hamberg K (2011). Swedish medical students’ expectations of their future life. Int J Med Educ.

[39] Diderichsen S, Johansson EE, Verdonk P, Lagro-Janssen T, Hamberg K (2013). Few gender differences in specialty preferences and motivational factors: a cross-sectional Swedish study on last-year medical students. BMC Med Educ..

[40] Dorsey ER, Jarjoura D, Rutecki GW (2005). The influence of controllable lifestyle and sex on the specialty choices of graduating U.S. medical students, 1996-2003. Acad Med.

[41] United Nations Development Programme (UNDP). Human development report 2016. Human development statistical tables. Table 5: gender inequality index. http://hdr.undp.org/sites/default/files/2016_human_development_report.pdf. Accessed 2 July 2018.

